# NOD-like Receptors in the Eye: Uncovering Its Role in Diabetic Retinopathy

**DOI:** 10.3390/ijms21030899

**Published:** 2020-01-30

**Authors:** Rayne R. Lim, Margaret E. Wieser, Rama R. Ganga, Veluchamy A. Barathi, Rajamani Lakshminarayanan, Rajiv R. Mohan, Dean P. Hainsworth, Shyam S. Chaurasia

**Affiliations:** 1 Ocular Immunology and Angiogenesis Lab, University of Missouri, Columbia, MO 652011, USA; rayne.ruiyi.lim@gmail.com (R.R.L.); mel7zd@health.missouri.edu (M.E.W.); mohanr@missouri.edu (R.R.M.); 2 Department of Biomedical Sciences, University of Missouri, Columbia, MO 652011, USA; 3 Ophthalmology, Harry S. Truman Memorial Veterans’ Hospital, Columbia, MO 652011, USA; 4 Surgery, University of Missouri, Columbia, MO 652011, USA; gangar@health.missouri.edu; 5 Singapore Eye Research Institute, Singapore 169856, Singapore; amutha.b.veluchamy@seri.com.sg (V.A.B.); lakshminarayanan.rajamani@seri.com.sg (R.L.); 6 Mason Eye Institute, School of Medicine, University of Missouri, Columbia, MO 652011, USA; HainsworthD@health.missouri.edu

**Keywords:** NOD-like receptors, NLRP3 inflammasome, ocular tissues, innate immune system, diabetic retinopathy, inflammation, retina

## Abstract

Diabetic retinopathy (DR) is an ocular complication of diabetes mellitus (DM). International Diabetic Federations (IDF) estimates up to 629 million people with DM by the year 2045 worldwide. Nearly 50% of DM patients will show evidence of diabetic-related eye problems. Therapeutic interventions for DR are limited and mostly involve surgical intervention at the late-stages of the disease. The lack of early-stage diagnostic tools and therapies, especially in DR, demands a better understanding of the biological processes involved in the etiology of disease progression. The recent surge in literature associated with NOD-like receptors (NLRs) has gained massive attraction due to their involvement in mediating the innate immune response and perpetuating inflammatory pathways, a central phenomenon found in the pathogenesis of ocular diseases including DR. The NLR family of receptors are expressed in different eye tissues during pathological conditions suggesting their potential roles in dry eye, ocular infection, retinal ischemia, cataract, glaucoma, age-related macular degeneration (AMD), diabetic macular edema (DME) and DR. Our group is interested in studying the critical early components involved in the immune cell infiltration and inflammatory pathways involved in the progression of DR. Recently, we reported that NLRP3 inflammasome might play a pivotal role in the pathogenesis of DR. This comprehensive review summarizes the findings of NLRs expression in the ocular tissues with special emphasis on its presence in the retinal microglia and DR pathogenesis.

## 1. Introduction

Aberrant inflammatory pathways constitute a major underlying mechanism in the perpetuation of several ocular diseases, including diabetic retinopathy (DR) [1,2]. While inflammation is considered as the first-response defensive process by the innate immune system, chronic or unregulated inflammation can result in excessive production of reactive oxidative species (ROS), proteases, growth factors and pro-inflammatory cytokines by innate immune cells [3]. This phenomenon leads to an autoinflammatory feedback loop, which mediates a pattern of positive feedback reinforced through tissue damage [4,5,6].

In retinal diseases, chronic inflammation is thought to stem from the non-microbial stimulus or sterile inflammation [1,2] due to the immune-privileged nature of the eye [7,8,9]. The blood-retinal barrier (BRB) protects the retina from inflammation-mediated damage supported by an apparent lack of a lymphatic system [10], and extensive-expression of immune modulators by retinal cells [7,9,11]. Multiple mechanisms are currently being studied to determine the molecular pathways involved during retinal inflammation [2,12,13], including inflammasome [14]. Moreover, pro-inflammatory cytokines such as IL-1β, IL-6, IL-8, and TNFα are released, which exacerbates sterile inflammation in DR [15,16,17].

DR is the leading cause of irreversible blindness amongst working adults in developed countries [18,19]. Recent data estimates an increasing trend of up to 629 million adults (aged 20–79 years) with diabetes mellitus (DM) by the year 2045 [20]. Currently, approximately 9.4% of the U.S. population is diabetic [21], of which one-third of DM patients are expected to exhibit some form of DR [20]. This number is expected to triple by 2050, with a pronounced increase among those 65 years and above [22]. Regardless of age at DM onset, statistics show that DM patients generally develop DR within ten years of diagnosis [18,23]. Thus, there is an urgent need to understand DR etiology and possible intervention during the early stages of the disease progression.

DR is a chronic low-grade inflammatory disease that causes gradual degeneration of the microvasculature, ultimately leading to the neural retinal dysfunction [24]. DR is broadly classified into two stages: Non-Proliferative (NPDR) and Proliferative (PDR). Diabetic macular edema (DME) also occurs during DR progression when fluid leaks into the macula, causing swelling and blurred vision [25]. Features of NPDR are mild and difficult to detect, with the earliest visible signs being microaneurysm formation and retinal hemorrhage [26]. Progressive occlusion in the retina leads to pericyte cell loss and accumulation of acellular capillaries [27], intraretinal vascular abnormalities and altered blood flow [28], and ultimately BRB breakdown [29]. PDR occurs with retinal ischemia that results in microvascular endothelial proliferation, vascular leakage, hemorrhage, and neural cell death [30].

Treatments for DR are limited and generally performed at the advanced stages of the disease, targeted mostly to minimize further damage to the vision [31]. Laser photocoagulation, intravitreal anti-VEGF injections, and vitreoretinal surgery are common DR interventions [32,33]. Multiple drugs targeting other systems involved in DR progression are also being tested currently for better management of the disease [34]. Nonetheless, their limited success urges the need for a more in-depth understanding of molecular pathways, which could exacerbate inflammation during DR [35].

## 2. Nucleotide-Binding and Oligomerization Domain (NOD)-like Receptors (NLRs)

**Figure 1:**
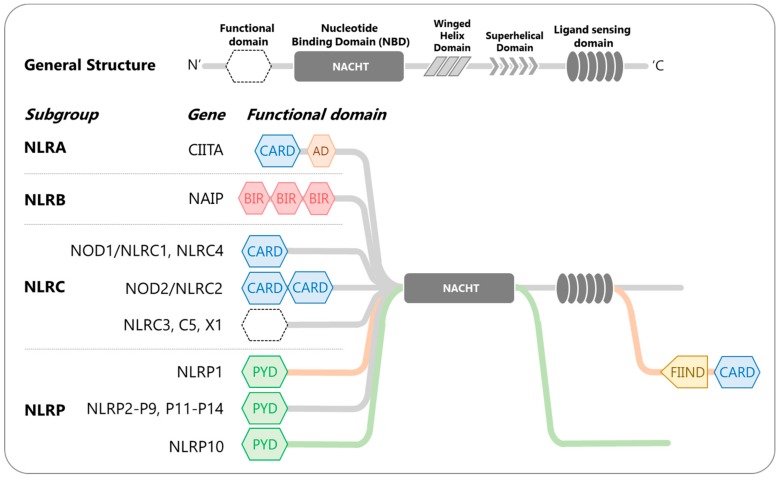
Human NOD-like receptors (NLRs)-classification and domain organization. The NLR proteins contain three distinct domains: (1) C-terminal LRRs for ligand sensing, (2) central NACHT for oligomerization, and (3) N-terminal effector domain to mediate signal transduction. Their functional domain structure further subdivided NLRs into four subfamilies: NLRA, NLRB, NLRC and NLRP. Legends: LRR—leucine-rich repeats; NACHT—NAIP, CIITA, HET-E, and TP1 proteins; CARD—caspase activation and recruitment domain; AD—activation domain; BIR—baculovirus IAP repeat; PYD—pyrin domain.

The NLRs are multi-domain, cytosolic receptors that form signaling complexes to mediate major cellular pathways [36]. Structurally tripartite, they contain three distinct domains: ligand-sensing leucine-rich repeats (LRRs) thought to be involved in pathogen sensing, a NACHT domain for oligomerization, and an effector domain at their N-terminal to mediate signal transduction. The NLRs are further divided into sub-categories based on the effector domains, (i) NLRC or NOD with caspase activation and recruitment domain (CARD); (ii) NLRP or NALP with pyrin domain (PYD); (iii) NLRB or NAIP with baculovirus IAP repeat (BIR) domain; and (iv) CIITA or NLRA with acidic transcriptional activation (TA) domain. Several reviews have attempted to categorize and define members within each subfamily, however, there is no universally accepted classification system yet. Nevertheless, there are 22 NLRs defined in humans, including 14 NLRPs, 6 NLRCs, 1 NLRB, and 1 CIITA [36,37,38], as illustrated in Figure 1.

Due to their numerous members and various structures, NLRs are versatile and participate in diverse biological processes. They are involved in innate and adaptive immunity via regulation of antigen presentation and differentiation of the adaptive immune response; host defense mechanism via recognition of pathogen-associated molecular patterns (PAMPS); inflammation modulation via signal transduction through nuclear factor-kappa B (NF-κB) and formation of inflammasome; regulation of cell death by monitoring metabolic sensors and damage-associated molecular patterns (DAMPS). As a critical component of many pathways, NLRs are activated by a wide variety of pathogenic, environmental, and endogenous signals. Mechanisms through which each NLR recognizes the range of signals are still not well understood.

NLRs have a role in embryonic development [39,40,41], in which the mutation of NLRP5 [42] and NLRP7 causes embryonic lethality. Zhang et al. reported the expression of NLRP4, NLRP5, NLRP8, NLRP9, NLRP11, NLRP12, NLRP13, and NLRP14 elevated in preimplantation human embryos [43]. Mutations in NLRs are associated with many human diseases. A frame-shift mutation in NOD2 results in a defective response to microbiota in the intestinal tract, leading to Crohn’s disease, an inflammatory bowel disease [44]. However, the role of NLR in eye development is yet to be studied. The current literature suggests that CIITA (otherwise known as major histocompatibility complex (MHC)-II transactivator) acts as a master switch for the expression of MHC-II genes [45], while NLRC5 regulates expression of MHC-I genes [46]. NLRB, also known as NAIP, responds to bacterial flagellin and is suggested to modulate NLRC4 inflammasome activation [47,48]. PAMP recognition, specifically for microbial peptidoglycan, is exceptionally well-characterized in NOD1 and NOD2 [49]. NODs rely on a common downstream adaptor molecule RIP2 for NF-κB activation [50], while NLRPs commonly form large signaling complexes with adaptor proteins and caspases called inflammasome [51], and also activates downstream NF-κB signaling pathway. NLRP recognizes both PAMPS and DAMPS, with NLRP3 being the most well-characterized member of the family. It is reported to be activated by uric crystals in gout [52], calcium phosphate crystals in osteoarthritis [53], amyloid-β oligomers in Alzheimer’s [54], and fatty acids in type 2 diabetes [55].

**Figure 2:**
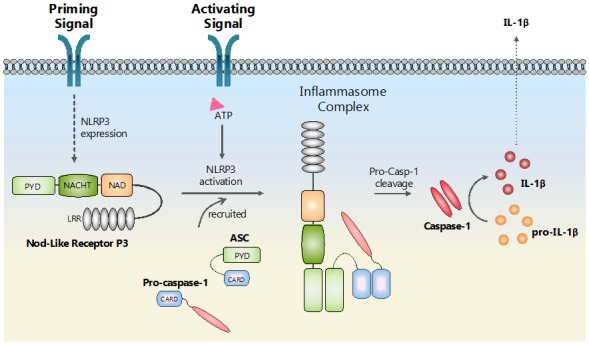
Schematic presentation of NLRP3 inflammasome activation.

Inflammasomes are of particular interest in ocular diseases due to their involvement in inflammatory pathways. These large protein complexes comprise of an NLR, an adaptor, and a recruited caspase-1. The NLRs oligomerizes following a stimulus, adopting the conformation thought to be similar to that of the apoptosome. NLRP3 is the most studied NLR, which requires a two-step triggering event for the activation process. A “priming” signal first activates NF-κB to induce NLRP3 and pro-IL-1β gene expression, followed by a second signal that activates the inflammasome. Typically, the effector domain, PYD, in NLRP inflammasomes contains a death fold, which recruits an adaptor protein—ASC (apoptosis speck-like protein containing a CARD)—through homotypic interactions. The CARD domain, in turn, recruits pro-caspase-1, resulting in close proximity to autocatalytic cleavage forming mature caspase-1. Proteolytic cleavage of downstream targets—pro-IL-1β and pro-IL-18, release active IL-1β, and IL-18 to mediate the inflammatory response in a paracrine fashion [56] (Figure 2). NLRP1 [56], NLRP3, 6 [57], NLRP7 [58], NLRP12 [59], and NLRC4 [60] have all been shown to activate the inflammasome. However, the activators for each NLR member are varied and seemingly limitless. Due to their intracellular localization, most NLRs are activated indirectly by external stimuli.

The versatility of the NLR system proves to be both advantageous and challenging. The wide range of activating ligands does not guarantee the same effect in different cell systems. For example, cholesterol crystals in atherosclerosis were found to activate the NLRP3 inflammasome in phagocytes/macrophages [61,62] however, failed to do the same in retinal pigment epithelium [63]. Similarly, high mobility group box-1 (HMGB-1) did not activate NLRP3 in THP-1 macrophages [64] but primed the inflammasome in the hippocampal microglial cells [65]. HMGB1 is an extracellular ligand that acts as an alarmin, mediating the increase in NF-κB, and TNFα/VEGF expression in human retinal pigment epithelial (ARPE-19) cells treated with high glucose [66]. Hence, unique localization and activation of NLRs in different retinal cell types and their function needs further studies for an accurate depiction of retinal diseases.

## 3. NOD-like Receptors in the Ocular Tissues

NLRs have been implicated in various ocular diseases such as ocular infection, dry eye, glaucoma, retinal ischemia, age-related macular degeneration (AMD), and DR. Table 1 summarizes the expression of NLRs in various ocular tissues.

The expression of NLRs on the ocular surface was first described in the human corneal epithelium by Benko et al. [67]. The group analyzed human epithelial tissue to show expression of NOD1, NOD2, NLRC5, and NLRX1 mRNA, which increased after peptidoglycan stimulation [67]. Subsequently, NOD1, NOD2, NLRP1, NLRP3 were shown to be regulated during bacterial or fungal infections in humans [68,69,70] and mouse [71,72] cornea. However, several reports suggested the source of the NLR proteins to be derived from infiltrated neutrophils or monocytes at the site of infection [73,74,75]. In dry eye conditions, human patients with environment-induced dry eye [76], murine models of dry eye [77,78], and alkali-burned mice [79] all showed increased mRNA levels of NLRP3 in their conjunctival epithelium. Corneal epithelial cells likewise increased NLRP3 levels under hyperosmotic stress, which was reduced by inhibitors [76,77,80,81]. Limited literature is available on the stroma and endothelium in the cornea. Primary corneal fibroblast cells expressed NOD1 and NOD2 [82], elevated NLRC4 after bacterial infection [83], but reduced NLRP1, NLRP3, NOD1, and NOD2 mRNA levels following LPS stimulation [68]. In contrast, corneal endothelial cells showed a significant increase of NLRP3 and NOD2 levels [68]. Corneal fibroblasts have well-established roles in mediating corneal fibrosis after insult [84] and are gaining attraction as immune modulators during infections [85]. In the conjunctiva, NLRP3 was observed at constitutive levels in healthy humans [86,87] and rat [87] tissues. Diseases such as Mooren’s ulcer [86] and pterygium [88] were found to elevate NLRP3 levels. Cultured human and rat goblet cells were also reported to increase NLRP3 significantly following bacterial infection [87]. Interestingly, NLRP3 in human goblet cells was shown to be activated only by the pathogenic bacteria, indicating a discriminative function towards external insult [89].

Few NLR studies have been reported on the experimental animals other than rodents. Immunohistochemistry on healthy canine corneas demonstrated NOD1 and NOD2 protein expression in the corneal epithelium, corneal endothelium, conjunctival epithelium, with scattered expression in the substantia propria [90]. Similar staining was also seen in the healthy mouse [90], indicating the conserved expression and localization in the ocular tissue across species.

In models of acute glaucoma, retinal ischemic-reperfusion (I/R) injury caused by elevated intraocular pressure (IOP) [91,92,93,94], optic nerve clamp [95], or optic nerve crush [96] resulted in upregulation of NLRP1 and NLRP3 in the retina. The resulting retinal ganglion cell loss was ameliorated in the *Nlrp3^−/−^* mouse [97]. Likewise, the NLRP3 increase was recapitulated in the D2 mouse model with an age-dependent increase in IOP [98]. Reduction in NLRP3 was seen in the arginase-2 knockout mouse [99], as well as with glycyrrhizic acid [100] and Kaempferol [101] treatment after I/R injury. Meanwhile, the mouse model of neovascular glaucoma depicted NOD2 downregulation in the whole cornea after arylsulfonyl indoline-benzamide treatment [102].

NLR regulation in retinal degeneration was demonstrated using multiple murine models. Light-induced retinopathy showed an increase in NLRP3 after one-month [103], which was alleviated by the deletion of Ccr2 [103], and treatment with monomethyl fumarate [104]. NMDA-induced retinal excitotoxicity also resulted in a time-dependent increase of retinal NLRP3 mRNA [105], while TXNIP knockout in Müller cell culture prevented NLRP3 production [106]. Meanwhile, a transgenic mouse with P23H on *Nlrp3^−/−^* background had reduced cone cell death [107]. A similar increase in NLRP3 transcripts was seen in canine models of human retinitis pigmentosa [108]. Furthermore, wild-type pups in models of oxygen-induced retinopathy also showed high levels of retinal NLRP1 [109] and NLRP3 [110,111] by postnatal day 10.

Most of the NLR literature in the retina comes from studies on AMD [150], where NLRP3 was found to be localized in the AMD lesions as well as the RPE and choroid of the patients with geographic atrophy (GA) and neovascular AMD [117,129,130]. Mice models of AMD including Ccl2/Cx3cr1 double knockout [117], chimeric Cfh transgenic [118], and Ceruloplasmin/Hephaestin double knockout (*Cpc^−/−^Heph^−/−^*) [132] similarly showed NLRP3 expression with age, while NLRP3 knockout in the *VEGF-A^hyper^* mouse resulted in a reduced number of choroidal neovascularization (CNV) lesions [133]. AMD drusen components such as amyloid-β [115], complement factor C1Q, carboxyethylpyrrole [151], and major lipofuscin component *N*-retinylidene-*N*-retinylethanolamine (A2E) [152], have been shown to prime and activate the NLRP3 inflammasome. Therefore, it is not surprising that intravitreal injection of Aβ 1-40 into rodent eyes caused an elevation of NLRP3 transcript in the neural retina and RPE/Choroid [115,116]. In this model, liver X receptor agonist (TO90) [116] as well as Vinpocetine [131] treatment attenuated NLRP3 production in the retina. Interestingly, *Nlrp3^−/−^* mice were reported to be protected against RPE degeneration following sub-retinal delivery of Alu RNA plasmid [130] or iron overload [132]. On the other hand, Doyle et al. found CNV lesions to be larger in *Nlrp3^−/−^* mice, as compared to wild-type counterparts [151]. Hence, it is likely that NLRP3 plays a dual role in retinal diseases.

Multiple pathways, including inflammation and oxidative stress, have been reported in AMD pathogenesis. In vitro, stimulation of RPE cells with LPS + TCDD (oxidative stress and low-grade inflammation), TNFα (inflammatory stress) [117], IL-1α (prime inflammasome components) [129,149], IL-17A (signature cytokine of Th17 cells) [136], 4-hydroxyhexenal (unsaturated aldehydes) [139], sodium iodate (NaIO_3_; oxidative stress) [142], oxidized-low density lipoprotein (ox-LDL; modified lipoprotein) [138,145], C5a (complement factor) [140], Aβ 1-40 [144,146], all resulted in elevated NLRP3 expression. Meanwhile, NLRP3 knockdown in A2E-treated ARPE-19 cells showed reduced ASC complex formation and IL-1β production [152]. Successful inhibition of NLRP3 was shown via inhibition of MAPK [146] and NF-κB [137] signaling pathways, as well as via treatments with cyanidin-3-glucoside [139] and puerarin—an antioxidant and anti-inflammatory compound [144].

## 4. NLRs in Diabetic Retinopathy

Several mechanistic studies have investigated the pathology and progression of DR. Hyperglycemia is recognized as the major factor in driving the regulation of various adverse responses in DR. Oxidative stress from hyperglycemia has been postulated to cause biochemical changes and activation of pathogenic mechanisms in the retina [153] via mitochondrial dysfunction and overproduction of ROS [154]. Therefore, overexpression of mitochondrial superoxide dismutase (SOD) was shown to prevent glucose-induced oxidative stress and VEGF expression [155] in DR development [156]. An increase in polyol pathway flux was also reported during mitochondrial dysregulation, in which glucose was reduced to sorbitol and increased advanced glycation end products (AGEs) formation, resulting in the exacerbation of oxidative stress [157]. This is usually accompanied by elevated production of inflammatory mediators such as IL-1β [158] and VEGF [159] and chemokines such as CCL2 [160], which are involved in the alteration of the BRB in the inner retina.

Chronic inflammation plays a central role in DR pathology [1,2]. Knockdown of CD18 and ICAM-1 involved in leukostasis reduces DR features, especially early indicators such as retinal occlusion and endothelial cell damage [161]. Inhibition of the IL-1β pathway also prevented the development of DR in diabetic animals [162]. Several inflammatory pathways, including those mediated by the NLRs, converge on NF-κB, the transcription factor involved in multiple critical regulatory pathways of proliferation, immune response, apoptosis [36], and angiogenesis [163]. NF-κB is a known regulator of IL-1β expression, which is processed to its matured form by NLRP3 [150]. As a result, attenuation of NF-κB via inhibition of its coactivator, poly(ADP-ribose) polymerase (PARP) [164], effectively reduced early retinal changes [165]. NF-κB is also known to modulate the activation of the NLRP3 inflammasome via regulating its expression [166,167]. Conversely, the role of NLRs in the regulation of inflammation and angiogenesis is not yet fully understood. Some members of the NLR family, including NOD1 and NOD2 were reported to activate NF-κB [168,169], while other members, including NLRP2 [170], NLRP6 [171,172], NLRP12 [173,174], NLRC3 [173] and NLRX1 [175] were reported to negatively regulate NF-κB signaling. However, the inhibitory functions of these NLRs are not well understood [176].

The prevalence and activity of NLRP3 have garnered significant interest across multiple disciplines involving inflammation. NLRP3 gain-of-function mutation results in excessive release of IL-1β, giving rise to cryopyrin-associated periodic syndrome (CAPS), a group of rare hereditary autoinflammatory diseases [4]. Several reports have shown evidence of ocular responses, including optic disc swelling [177], uveitis, keratitis, and conjunctivitis [178] in CAPS patients. Treatment of CAPS with anakinra, an interleukin-1 receptor antagonist, alleviates symptoms associated with autoinflammation [179]. In DR, caspase-1, and IL-1β were found in both retinas and vitreous of diabetic patients [180,181]. In addition, caspase-1/IL-1β dependent cell death, also described as pyroptosis, has been implicated in the cell death of retinal cells, including Müller and microglial cells [182]. However, despite the role of NLRs in the regulation of caspase-1 and IL-1β, its involvement in pyroptosis has not yet been elucidated. Limited literature was reported in regard to the specific impact of NLRs on neuronal or microvascular cell death in DR. The first direct link between NLRP3 and endothelial cell death in the retina was suggested in ischemic retinopathy, where hypoxia upregulated NLRP3 and IL-1β expression in rat retinal microglia [111]. While IL-1β was not directly cytotoxic to the endothelial cell, IL-1β-induced production of Sema3A in retinal ganglion cells led to endothelial cell death by activated caspase-3 [111]. Studies are further hampered by the insufficiency of many animal models, which limit the clarification of NLR expression in different stages of the disease.

In human DR patients, retinal proliferative membranes obtained from PDR patients during vitreoretinal surgery showed a significant increase in NLRP3 expression, which were co-localized with CD31+ endothelial cells [120]. Using rodent models of DR, STZ-induced diabetic mice showed DR features accompanied by increased retinal NLRP1 [109] and NLRP3 [123] expression. Similarly, STZ-induced diabetic rats also had elevated retinal NLRP3 protein, which was attenuated by sulforaphane [121] and methylene blue [122] treatment. Diabetic *Nlrp1^−/−^* mice displayed reduced severity of DR [109], while fenofibrate given to STZ-wild-type mice reduced NLRP3 activation and ameliorated retinal vascular leakage [123]. Meanwhile, the Akimba mouse model of PDR showed no changes in NLRP1 expression but a significant increase in NLRP3 protein across the inner retina [114]. In a type 2 diabetic model, rats fed a high-fat diet for ten weeks showed increased NLRP3 in the whole retina, which was later found to immunoprecipitate with thioredoxin-interacting protein (TXNIP) in human retinal endothelial cells [113]. Taken together, in vivo data suggests NLRP3 to play significant roles in perpetuating inflammation in the retinal tissue during DR.

Numerous cells are implicated in the progression of DR. Müller cells are specialized radial cells that span the height of the retina, providing both structural support and metabolic regulation of the tissue, including production of inflammatory cytokines. Evidence demonstrating the activation of retinal microglia and Müller cells in the early stages of diabetes contributes a major role in the onset of inflammatory processes. In vitro, primary rat Müller cells treated with 25 mM glucose had elevated NLRP3 after two days, which was reduced with sulforaphane treatment [121]. Likewise, immortalized Müller cell line, rMC1, treated with 25 mM glucose expressed maximal NLRP3 protein three days after high-glucose induction, with minimal changes within the first 24 h [125]. Retinal ganglion cells (RGCs) are output neurons which relay visual signals from the retina to the brain visual centers. Mice RGCs treated with 5 mM fructose upregulated NLRP1 expression after 24 h [109], while rat RGCs stimulated with 20 mM glucose for 48 h upregulated NLRP3 expression [127]. Inhibition of TLR4 using TAK-242 was found to attenuate NLRP3 and IL-1β production in the rat RGCs [127]. Retinal microvascular endothelial cells form the crucial inner BRB that shields the inner retina from circulatory insult. The primary human retinal microvascular endothelial cells (HRMECs) treated with 30 mM glucose elevated NLRP3 expression, but was not reduced by the NLRP3 inhibitor, MCC950 [120]. Instead, MCC950 downregulated NEK7-NLRP3 interaction, which led to a reduction in IL-1β production [120]. Epac-1 agonist similarly reduced NLRP3 mRNA levels in HRMECs treated with 25 mM glucose [128]. Retinal pigmented epithelial (RPE) cells form the outer BRB that regulates material transfer between the choroidal circulation and the photoreceptors. Immortalized ARPE-19 cells treated with a combination of 15 mM glucose + IL-1β + TNFα for 24 h resulted in increased NLRP3 stained puncta in the cells, which were reduced when treated with connexin 43 hemichannel blocker (Peptide5) [143]. Likewise, ARPE-19 treated with 30 mM glucose upregulated NLRP3 production, which was exacerbated when autophagy was inhibited by 3-methyadenine (3-MA) [147]. All these studies demonstrated the ubiquitous expression of NLRP3 in several cell types of the retina, demonstrating key roles for the NLR proteins in the cellular mechanisms during DR pathogenesis.

Augmented mitophagy/autophagy could lead to mitochondrial (mt) dysfunction, which is one of the mechanisms implicated in DR progression [183]. Excessive ROS production is a hallmark of oxidative stress, and has been implicated extensively in NLRP3 activation with different initiators of damage [146,148]. Release of mtDNA during mitochondrial stress has a direct role in the activation of NLRP3 inflammasome [184], in turn causing additional mitochondrial instability [185]. Furthermore, enhanced mitophagic flux could cause lysosomal destabilization [186], with the subsequent release of cathepsin B [187], activating the inflammasome via increased ROS [129]. Autophagy was also proposed to be an inflammasome-priming regulator by the downregulation of NF-κB and other signaling components [188,189]. Indeed, blocking autophagy or proteasomal clearance in RPE cells resulted in increased secretion of NLRP3 into the culture media [141] and activation of NLRP3 inflammasome [149].

Recent reports on TXNIP elucidated the link between hyperglycemia, oxidative stress, inflammation, and cell death in diabetic retinopathy [190,191]. TXNIP is an inhibitor of thioredoxin, a major cellular antioxidant and anti-apoptotic protein ubiquitously expressed in all cells. It regulates glucose uptake in skeletal muscle cells by mediating insulin resistance [192]. TXNIP was shown to induce inflammation through direct binding to NLRP3 inflammasome [193], leading to caspase-1 activation [113,194] and IL-1β secretion. Under high glucose conditions, TXNIP levels were found to increase in the rat Müller cell line, along with enhanced mitophagy [195]. Similarly, high-glucose induced TXNIP activation in retinal endothelial and Müller glial cells were also shown to promote oxidative stress [196]. TXNIP knockdown subsequently restored LC3B levels and normalized mitophagic flux [195], indicating its role in regulating homeostasis in the retinal cells. Consequentially, the murine TXNIP knockout model showed reduced NLRP3 activation and retinal inflammation [197]. Besides, primary mouse Müller cells isolated from TXNIP knockout mouse also failed to upregulate NLRP3 after palmitate treatment [126]. Inhibition of TXNIP activity in vivo via siRNA-mediated transcriptional gene silencing (TGS) successfully attenuated inflammation and gliosis [106]. Given these promising data, the therapeutic potential of RNAi-mediated TGS in retinal treatment warrants further investigation.

**Figure 3:**
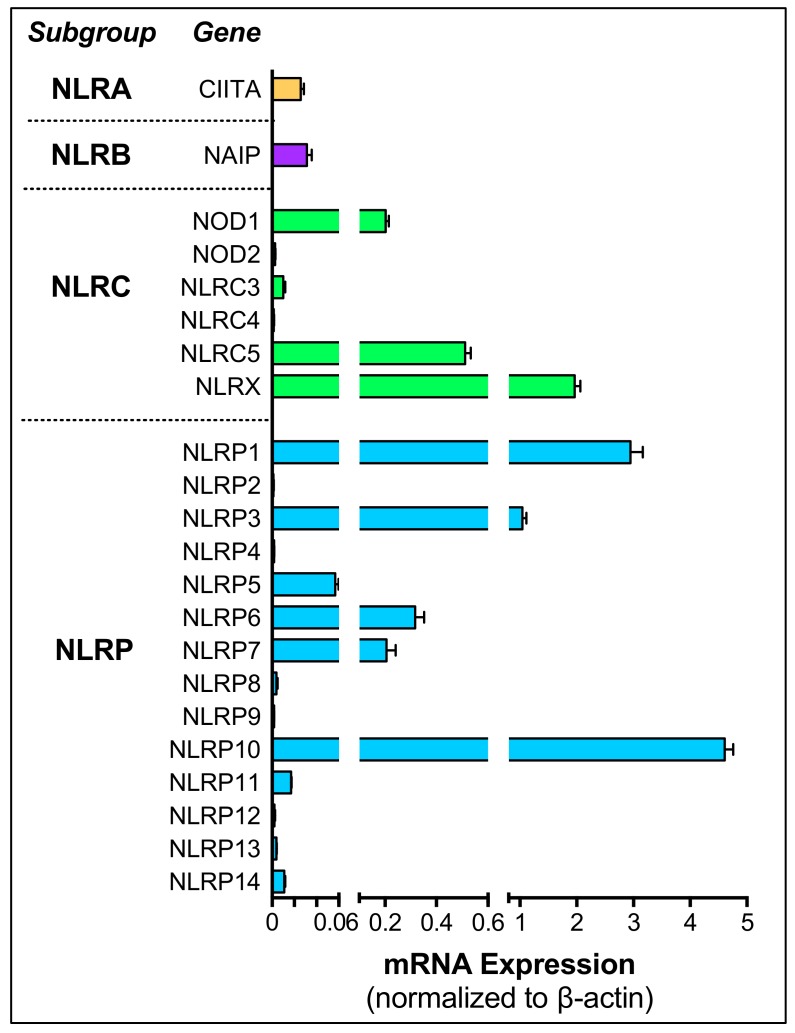
Expression profile of NLR genes in HMC3 microglia cells. Human brain microglial cell line (HMC3, ATCC^®^ CRL-3304^TM^) showed expression of NOD1, NLRC5, NLRX, NLRP1, NLRP3, NLRP6, NLRP7 and NLRP10. Real-time PCR was performed with *n* = 6 samples in duplicate, with β-actin as the housekeeping gene. The expression of each NLR family transcript was normalized relative to the NLRP3 expression levels. Data is represented as mean ± SEM.

Retinal microglial cells are garnering huge interest in DR pathogenesis due to their enormous capacity to orchestrate neuroinflammatory responses, as well as their roles as first responders during retinal insults [198]. Microglia were observed to be activated in human DR retina, typically exemplified by their transformation from ramified to amoeboid morphology, increase in cell number, as well as locational changes in the retina [199]. Recent clinical OCT imaging of human T2DM also found hyper-reflective spots located in the inner retina, described to be activated microglia accumulation [200]. In murine models, microglia were also found in increased numbers in the outer plexiform and ganglion cell layer four months after diabetes induction, which steadily increased with the progression of the disease [201]. During PDR, the Akimba mouse model also has Iba-1/CD11b positive cells that were accumulated in the inner retina, where NLRP3 expression was most robust [114]. Besides NLRP3, microglia were also found to express a comprehensive profile of NLRs. Using a human brain microglial cell line (HMC3), we found microglial cells to constitutively express NOD1, NLRC5, NLRX, NLRP1, NLRP3, NLRP6, NLRP7, and NLRP10 (Figure 3).

Microglia with NLRP3 activation was further implicated in other models of retinal damage. Rats that underwent optic nerve crush (ONC) heightened NLRP3 staining in Iba-1+ microglia cells that aggregated in the optic nerve one-day after injury [97]. Similarly, light-induced retinopathy showed NLRP3 co-localization in Iba-1+ retinal microglial cells after one month [103]. The rat P23H retinal degenerative model had CD11b+ retinal microglial cells co-stained with NLRP3 [107]. Cultured rat retinal microglial cells exposed to 80% O_2_ showed elevated NLRP3 expression, which was attenuated with NAC [111]. Immortalized mouse microglial cells (BV2) subjected to hypoxia also elevated NLRP3 production [92]. Meanwhile, microglia in the subretinal region of the AMD mouse model (CFH transgenic mouse) also showed NLRP3 staining [118]. Subsequently, retinal microglial cells primed with LPS and stimulated with 7-ketocholesterol (a component of lipoprotein deposits) showed an increased number of NLRP3 stained puncta in the cytoplasm [124]. Similarly, primary pig retinal microglia (pMicroglia) cultured in our lab significantly upregulated IL-1β following LPS insult [202]. These reports indicate the responsiveness of microglia to a retinal insult, and suggest the NLRP3 inflammasome to play a pivotal role in mediating neuroinflammation and angiogenesis.

Successful treatment of DR requires a deep understanding of not only the molecular mechanisms involved in disease pathogenesis, but also the time-course development of each molecular player. Microglia and Müller cell activation are one of the earliest cellular responses during retinal insult, which has been reported to precede vascular or neuronal dysfunction. Since inflammatory cytokines such as IL-1β are commonly found in DR retina, the rationale follows that NLR activation might occur in the early stages of DR. A prolonged period of unmitigated and uncontrolled inflammation is involved in DR progression, thus early intervention in the NLR activation pathway could be beneficial in halting the disease. Pharmacological inhibition of microglia cells using Minocycline, while showed promising results in ameliorating DR presentation [203], has not yet been studied in context with the NLR proteins. A well-known inhibitor MCC950, binds to the NACHT domain of NLRP3 to prevent it from assuming an open conformation ready for ATP hydrolysis, was successful in preventing NLRP3 activation and HRMEC apoptosis [120]. Other NLRP3 specific inhibitors [204] include CY-09 [205], 3,4-Methylenedioxy-β-nitrostyrene, Tranilast [206], OLT1177 and Oridonin. Interestingly, Tranilast was reported to be effective in vitro in a three-drug combination, indicating the possibility of a combinatory therapy for maximum effectiveness in DR therapy.

## 5. Future Directions

The key to successful intervention is the timely application of therapeutic drugs during the progression of DR pathogenesis. Current limitations due to the unavailability of the DR phenotype restricts the intervention to the late stages of proliferative DR. The transgenic mice models presently used are often accelerated, selective, and are difficult to reproduce across laboratories. Development of large animal models such as pigs [207] will be advantageous for their ability to develop DR phenotype/progression comparable to humans. Additionally, it is critical to map the expression of NLR proteins in different DR stages, in order to develop new targets and better understand the optimal time for drug delivery. Specific NLRP3 inhibitors, or indirect inhibitors to the NLRP3 activation pathway need to be tested in the in vivo models to better understand its role in neuronal dysfunction, as well as its effects on cell death in endothelial and pericyte cells. Gene editing specific to microglial NLRP3 could be further useful for ameliorating the inflammatory cascade of events to delay/prevent DR progression. Lastly, much work on the retina has been focused on the NLRP3 alone due to its extensive characterization. Since multiple NLR family members are expressed in multiple cell types of the retina, it might also be worthwhile to understand their compensatory role during DR progression.

## 6. Conclusions

The ubiquity of various NLR family members in the ocular tissues highlighted the versatility of NLR responses to ocular insults, thereby making them critical regulators for the initiation of inflammation especially in the DR pathogenesis. Currently, there is paucity of data in relation to NLRs in DR from human studies. The retina is a highly complex neurosensory tissue with multiple cell types [208,209]. DR progression involves a myriad of cells across all layers of the retina, of which the microglia were shown to express multiple members to regulate inflammasomes. Not only are they found in several regulatory pathways, but they are expressed spatially and temporally based on the immune status of the cell. Retinal microglial cells present an exciting aspect during DR pathogenesis and targeting NLRs could provide a potential therapeutic intervention for the prevention of DR progression.

## Figures and Tables

**Table 1:** NOD-like receptors (NLRs) in Ocular Tissues.

Ocular Tissues/Cells	Human	Transcript/Protein	Ref	Rat	Transcript/Protein	Ref	Mouse	Transcript/Protein	Ref	Canine	Transcript/Protein	Ref
Cornea	NLRP1	mRNA	[68]	NLRP1	mRNA	-	NLRP1	mRNA	-	NLRP1	mRNA	-
	NLRP3	mRNA	[68]	NLRP3	mRNA	-	NLRP3	mRNA	[74,78,79]	NLRP3	mRNA	-
		Protein	-		Protein	-		Protein	[71,72,78]		Protein	-
	NLRC4	Protein	-	NLRC4	Protein	-	NLRC4	Protein	[72]	NLRC4	Protein	-
	NLRB	mRNA	-	NLRB	mRNA	-	NLRB	mRNA	[74]	NLRB	mRNA	-
	NOD1	mRNA	[68]	NOD1	mRNA	-	NOD1	mRNA	-	NOD1	mRNA	-
	NOD2	mRNA	[68]	NOD2	mRNA	-	NOD2	mRNA	[102]	NOD2	mRNA	-
		Protein	-		Protein	-		Protein	[102]		Protein	-
Corneal Epithelium	NLRP3	mRNA	-	NLRP3	mRNA	-	NLRP3	mRNA	[77]	NLRP3	mRNA	-
		Protein	-		Protein			Protein	[77,78,79]		Protein	-
	NOD1	mRNA	[67]	NOD1	mRNA	-	NOD1	mRNA	-	NOD1	mRNA	-
		Protein	-		Protein	-		Protein	[90]		Protein	[90]
	NOD2	mRNA	[67]	NOD2	mRNA	-	NOD2	mRNA	-	NOD2	mRNA	-
		Protein	-		Protein	-		Protein	[90]		Protein	[90]
	NLRC5	mRNA	[67]	NLRC5	mRNA	-	NLRC5	mRNA	-	NLRC5	mRNA	-
	NLRX1	mRNA	[67]	NLRX1	mRNA	-	NLRX1	mRNA	-	NLRX1	mRNA	-
Corneal Limbal Epithelial (cell culture)	NLRP1	mRNA	[68]	NLRP1	mRNA	-	NLRP1	mRNA	-	NLRP1	mRNA	-
	NLRP3	mRNA	[68]	NLRP3	mRNA	-	NLRP3	mRNA	-	NLRP3	mRNA	-
	NOD1	mRNA	[68]	NOD1	mRNA	-	NOD1	mRNA	-	NOD1	mRNA	-
	NOD2	mRNA	[68]	NOD2	mRNA	-	NOD2	mRNA	-	NOD2	mRNA	-
Corneal Epithelial (cell culture)	NLRP1	mRNA	[67]	NLRP1	mRNA	-	NLRP1	mRNA	-	NLRP1	mRNA	-
		Protein	[67]		Protein	-		Protein	-		Protein	-
	NLRP2	mRNA	[67]	NLRP2	mRNA	-	NLRP2	mRNA	-	NLRP2	mRNA	-
	NLRP3	mRNA	[67,76,77,80,81]	NLRP3	mRNA	-	NLRP3	mRNA	-	NLRP3	mRNA	-
		Protein	[76,77,80,81]		Protein	-		Protein	-		Protein	-
	NLRP6	mRNA	[77]	NLRP6	mRNA	-	NLRP6	mRNA	-	NLRP6	mRNA	-
		Protein	[77]		Protein	-		Protein	-		Protein	-
	NLRP7	mRNA	[67]	NLRP7	mRNA	-	NLRP7	mRNA	-	NLRP7	mRNA	-
	NLRP10	mRNA	[67]	NLRP10	mRNA	-	NLRP10	mRNA	-	NLRP10	mRNA	-
	NOD1	mRNA	[67,69]	NOD1	mRNA	-	NOD1	mRNA	-	NOD1	mRNA	-
		Protein	[69]		Protein	-		Protein	-		Protein	-
	NOD2	mRNA	[67,70]	NOD2	mRNA	-	NOD2	mRNA	-	NOD2	mRNA	-
		Protein	[70]		Protein	-		Protein	-		Protein	-
	NLRC4	mRNA	[67]	NLRC4	mRNA	-	NLRC4	mRNA	-	NLRC4	mRNA	-
	NLRX1	mRNA	[67]	NLRX1	mRNA	-	NLRX1	mRNA	-	NLRX1	mRNA	-
Corneal Fibroblast (cell culture)	NLRP1	mRNA	[68]	NLRP1	mRNA	-	NLRP1	mRNA	-	NLRP1	mRNA	-
	NLRP3	mRNA	[68]	NLRP3	mRNA	-	NLRP3	mRNA	-	NLRP3	mRNA	-
	NLRC4	Protein	[83]	NLRC4	Protein	-	NLRC4	Protein	-	NLRC4	Protein	-
	NOD1	mRNA	[68]	NOD1	mRNA	-	NOD1	mRNA	[82]	NOD1	mRNA	-
	NOD2	mRNA	[68]	NOD2	mRNA	-	NOD2	mRNA	[82]	NOD2	mRNA	
Corneal Endothelium	NOD1	Protein	-	NOD1	Protein	-	NOD1	Protein	[90]	NOD1	Protein	[90]
	NOD2	Protein	-	NOD2	Protein	-	NOD2	Protein	[90]	NOD2	Protein	[90]
Corneal Endothelial (cell culture)	NLRP1	mRNA	[68]	NLRP1	mRNA	-	NLRP1	mRNA	-	NLRP1	mRNA	-
	NLRP3	mRNA	[68]	NLRP3	mRNA	-	NLRP3	mRNA	-	NLRP3	mRNA	-
	NOD1	mRNA	[68]	NOD1	mRNA	-	NOD1	mRNA	-	NOD1	mRNA	-
		Protein	-		Protein	-		Protein	-		Protein	-
	NOD2	mRNA	[68]	NOD2	mRNA	-	NOD2	mRNA	-	NOD2	mRNA	-
Conjunctiva	NLRP3	mRNA	[86]	NLRP3	mRNA	-	NLRP3	mRNA	[78]	NLRP3	mRNA	-
		Protein	[86,87]		Protein	[87]		Protein	[78]		Protein	-
Conjunctival Epithelium	NLRP3	mRNA	[76,88]	NLRP3	mRNA	-	NLRP3	mRNA	[77]	NLRP3	mRNA	-
		Protein	[88]		Protein	-		Protein	[77,78]		Protein	-
	NLRP6	mRNA	-	NLRP6	mRNA	-	NLRP6	mRNA	[77]	NLRP6	mRNA	-
		Protein	-		Protein	-		Protein	[77]		Protein	-
	NOD1	Protein	-	NOD1	Protein	-	NOD1	Protein	[90]	NOD1	Protein	[90]
	NOD2	Protein	-	NOD2	Protein	-	NOD2	Protein	[90]	NOD2	Protein	[90]
Conjunctival Goblet Cell (cell culture)	NLRP3	mRNA	[89]	NLRP3	mRNA	[87]	NLRP3	mRNA	-	NLRP3	mRNA	-
		Protein	[87,89]		Protein	[87]		Protein	-		Protein	-
Conjunctival Substantia Propria	NOD1	Protein	-	NOD1	Protein	-	NOD1	Protein	[90]	NOD1	Protein	[90]
	NOD2	Protein	-	NOD2	Protein	-	NOD2	Protein	-	NOD2	Protein	[90]
Iris Vascular Endothelial Cells (primary culture)	NOD2	mRNA	[112]	NOD2	mRNA	-	NOD2	mRNA	-	NOD2	mRNA	-
Nonpigmented Ciliary Body Epithelium	NOD1	Protein	-	NOD1	Protein	-	NOD1	Protein	-	NOD1	Protein	[90]
	NOD2	Protein	-	NOD2	Protein	-	NOD2	Protein	-	NOD2	Protein	[90]
Retina	NLRP1	mRNA	-	NLRP1	mRNA	[91,95,113]	NLRP1	mRNA	[109,114]	NLRP1	mRNA	-
		Protein	-		Protein	[91,95]		Protein	[101,109]		Protein	-
	NLRP1b	mRNA	-	NLRP1b	mRNA	-	NLRP1b	mRNA	[96]	NLRP1b	mRNA	-
	NLRP3	mRNA	-	NLRP3	mRNA	[91,93,95,111,113,115]	NLRP3	mRNA	[93,96,98,100,104,105,114,116,117,118]	NLRP3	mRNA	[108]
		Protein	[119,120]		Protein	[91,95,107,113,121,122]		Protein	[92,94,98,99,100,101,103,110,114,116,123]		Protein	[108]
	NLRC4	mRNA	-	NLRC4	mRNA	[113]	NLRC4	mRNA	-	NLRC4	mRNA	-
Retinal Microglia	NLRP3	Protein	-	NLRP3	Protein	[107]	NLRP3	Protein	[97,103,118]	NLRP3	Protein	-
Retinal Microglia (cell culture)	NLRP3	mRNA	-	NLRP3	mRNA	[111]	NLRP3	mRNA	-	NLRP3	mRNA	-
		Protein	-		Protein	-		Protein	[92,124]		Protein	-
Retinal Müller (cell culture)	NLRP3	Protein	-	NLRP3	Protein	[121,125]	NLRP3	Protein	[106,126]	NLRP3	Protein	-
Retina Astrocytes	NLRP3	Protein	-	NLRP3	Protein	-	NLRP3	Protein	[94]	NLRP3	Protein	-
Retina Ganglion Cell	NLRP1	Protein	-	NLRP1	Protein	-	NLRP1	Protein	[94]	NLRP1	Protein	-
	NLRP3	Protein	-	NLRP3	Protein	-	NLRP3	Protein	[94]	NLRP3	Protein	-
Retina Ganglion Cell (cell culture)	NLRP1	Protein	-	NLRP1	Protein	-	NLRP1	Protein	[109]	NLRP1	Protein	-
	NLRP3	mRNA	-	NLRP3	mRNA	[127]	NLRP3	mRNA	-	NLRP3	mRNA	-
		Protein	-		Protein	[127]		Protein	-		Protein	-
Retina Microvascular Endothelial Cell (cell culture)	NLRP3	mRNA	[120]	NLRP3	mRNA	-	NLRP3	mRNA	-	NLRP3	mRNA	-
		Protein	[113,120,128]		Protein	-		Protein	-		Protein	-
	NOD2	mRNA	[112]	NOD2	mRNA	-	NOD2	mRNA	-	NOD2	mRNA	-
Retina Pigmented Epithelium	NLRP3	mRNA	[117]	NLRP3	mRNA	-	NLRP3	mRNA	-	NLRP3	mRNA	-
		Protein	[119,129,130]		Protein	[115,131]		Protein	[132,133]		Protein	
Retina Pigmented Epithelial Cell (primary culture)	NLRP2	mRNA	[134]	NLRP2	mRNA	-	NLRP2	mRNA	-	NLRP2	mRNA	-
	NLRP3	mRNA	[134,135,136,137,138]	NLRP3	mRNA	-	NLRP3	mRNA	[119,137]	NLRP3	mRNA	-
		Protein	[134,136,137]		Protein	-		Protein	[119]		Protein	-
Retina Pigmented Epithelial Cell (ARPE-19)	NLRP3	mRNA	[117,138,139,140,141]	NLRP3	mRNA	-	NLRP3	mRNA	-	NLRP3	mRNA	-
		Protein	[129,141,142,143,144,145,146,147,148,149]		Protein	-		Protein	-		Protein	-
Choroid	NLRP3	mRNA	-	NLRP3	mRNA	-	NLRP3	mRNA	[116]	NLRP3	mRNA	-
		Protein	[119,129]		Protein	[115]		Protein	[116]		Protein	-
Choroid Vascular Endothelium	NOD2	Protein	[112]	NOD2	Protein	-	NOD2	Protein	-	NOD2	Protein	-
Choroidal Vascular Endothelial Cell (primary culture)	NOD2	mRNA	[112]	NOD2	mRNA	-	NOD2	mRNA	-	NOD2	mRNA	-

## Abbreviations

AMD	Age-related macular degeneration
AGEs	Advanced glycation endproducts
ARPE-19	Human retinal pigment epithelial cell line
ASC	Apoptosis speck-like protein containing a CARD
BRB	Blood-retinal barrier
CAPs	Cryopyrin-associated periodic syndrome
CNV	Choroidal neovascularization
DAMPS	Damage-associated molecular patterns
DM	Diabetes mellitus
DR	Diabetic retinopathy
GA	Geographic atrophy
HMC3	Human brain microglia cell line
HRMEC	Human retinal microvascular endothelial cells
I/R	Ischemic/Reperfusion
IOP	Intraocular pressure
NLRs	NOD-like receptors
NOD	Nucleotide-Binding and Oligomerization Domain
NPDR	Non-Proliferative DR
ONC	Optic nerve crush
PAMPS	Pathogen-associated molecular patterns
PDR	Proliferative DR
RGCs	Retinal ganglion cells
ROS	Reactive oxygen species
TGS	Transcriptional gene silencing
TXNIP	Thioredoxin-interacting protein

## References

[1] Tang J., Kern T. (2011). Inflammation in Diabetic Retinopathy. Prog. Retin. Eye Res..

[2] Rübsam A., Parikh S., Fort P.E. (2018). Role of Inflammation in diabetic retinopathy. Int. J. Mol. Sci..

[3] Chen G.Y., Nuñez G. (2010). Sterile inflammation: Sensing and reacting to damage. Nat. Rev. Immunol..

[4] Kuemmerle-Deschner J.B. (2015). Caps—Pathogenesis, presentation and treatment of an autoinflammatory disease. Semin. Immunopathol..

[5] Zhang W., Liu H., Al-Shabrawey M., Caldwell R.W., Caldwell R.B. (2011). Inflammation and diabetic retinal microvascular complications. J. Cardiovasc. Dis. Res..

[6] Wooff Y., Man S.M., Aggio-Bruce R., Natoli R., Fernando N. (2019). IL-1 Family Members Mediate Cell Death, Inflammation and Angiogenesis in Retinal Degenerative Diseases. Front. Immunol..

[7] Xu H., Chen M. (2017). Diabetic retinopathy and dysregulated innate immunity. Vision Res..

[8] Ferguson T.A., Griffith T.S. (1997). A vision of cell death: Insights into immune privilege. Immunol. Rev..

[9] Forrester J.V., Xu H. (2012). Good news-bad news: The Yin and Yang of immune privilege in the eye. Front. Immunol..

[10] Nakao S., Hafezi-Moghadam A., Ishibashi T. (2012). Lymphatics and lymphangiogenesis in the eye. J. Ophthalmol..

[11] Wenkel H., Streilein J.W. (2000). Evidence that retinal pigment epithelium functions as an immune-privileged tissue. Investig. Ophthalmol. Vis. Sci..

[12] Abcouwer S.F. (2011). Neural inflammation and the microglial response in diabetic retinopathy. J. Ocul. Biol. Dis. Infor..

[13] Lim R.R., Vaidya T., Gadde S.G., Yadav N.K., Sethu S., Hainsworth D.P., Mohan R.R., Ghosh A., Chaurasia S.S. (2019). Correlation between systemic S100A8 and S100A9 levels and severity of diabetic retinopathy in patients with type 2 diabetes mellitus. Diabetes Metab. Syndr. Clin. Res. Rev..

[14] Yerramothu P., Vijay A.K., Willcox M.D.P. (2018). Inflammasomes, the eye and anti-inflammasome therapy. Eye.

[15] Huang H., Gandhi J.K., Zhong X., Wei Y., Gong J., Duh E.J., Vinores S.A. (2011). TNFα Is Required for Late BRB Breakdown in Diabetic Retinopathy, and Its Inhibition Prevents Leukostasis and Protects Vessels and Neurons from Apoptosis. Investig. Opthalmology Vis. Sci..

[16] Funatsu H., Noma H., Mimura T., Eguchi S., Hori S. (2009). Association of Vitreous Inflammatory Factors with Diabetic Macular Edema. Ophthalmology.

[17] Vujosevic S., Simó R. (2017). Local and systemic inflammatory biomarkers of diabetic retinopathy: An integrative approach. Investig. Ophthalmol. Vis. Sci..

[18] Yau J.W.Y., Rogers S.L., Kawasaki R., Lamoureux E.L., Kowalski J.W., Bek T., Chen S.J., Dekker J.M., Fletcher A., Grauslund J. (2012). Global prevalence and major risk factors of diabetic retinopathy. Diabetes Care.

[19] Ding J., Wong T.Y. (2012). Current Epidemiology of Diabetic Retinopathy and Diabetic Macular Edema. Curr. Diab. Rep..

[20] International Diabetes Federation (2017). IDF Diabetes Atlas.

[21] Centers for Disease Control and Prevention (2017). National Diabetes Statistics Report, 2017.

[22] Saaddine J.B., Honeycutt A.A., Narayan K.M.V., Zhang X., Klein R., Boyle J.P. (2008). Projection of diabetic retinopathy and other major eye diseases among people with diabetes mellitus: United States, 2005–2050. Arch. Ophthalmol..

[23] Jones C.D., Greenwood R.H., Misra A., Bachmann M.O. (2012). Incidence and Progression of Diabetic Retinopathy During 17 Years of a Population-Based Screening Program in England. Diabetes Care.

[24] Kern T.S. (2007). Contributions of inflammatory processes to the development of the early stages of diabetic retinopathy. Exp. Diabetes Res. iabetes Res..

[25] Cheung N., Mitchell P., Wong T.Y. (2010). Diabetic Retinopathy. Lancet.

[26] Wilkinson C.P., Ferris F.L., Klein R.E., Lee P.P., Agardh C.D., Davis M., Dills D., Kampik A., Pararajasegaram R., Verdaguer J.T. (2003). Proposed international clinical diabetic retinopathy and diabetic macular edema disease severity scales. Ophthalmology.

[27] Kern T.S., Engerman R.L. (1995). Vascular lesions in diabetes are distributed non-uniformly within the retina. Exp. Eye Res..

[28] Curtis T.M., Gardiner T.A., Stitt A.W. (2009). Microvascular lesions of diabetic retinopathy: Clues towards understanding pathogenesis?. Eye.

[29] Durham J.T., Herman I.M. (2011). Microvascular modifications in diabetic retinopathy. Curr. Diab. Rep..

[30] Cai J., Boulton M. (2002). The pathogenesis of diabetic retinopathy: Old concepts and new questions. Eye.

[31] Stewart M.W. (2016). Treatment of diabetic retinopathy: Recent advances and unresolved challenges. World J. Diabetes.

[32] Gross J.G., Glassman A.R., Jampol L.M., Inusah S., Aiello L.P., Antoszyk A.N., Baker C.W., Berger B.B., Bressler N.M., Browning D. (2015). Panretinal photocoagulation vs. intravitreous ranibizumab for proliferative diabetic retinopathy: A randomized clinical trial. JAMA.

[33] Oellers P., Mahmoud T.H. (2016). Surgery for proliferative diabetic retinopathy: New tips and tricks. J. Ophthalmic Vis. Res..

[34] Bolinger M.T., Antonetti D.A. (2016). Moving past anti-VEGF: Novel therapies for treating diabetic retinopathy. Int. J. Mol. Sci..

[35] Zorena K. (2014). Anti-Inflammatory Therapy in Diabetic Retinopathy. Mediators Inflamm..

[36] Motta V., Soares F., Sun T., Philpott D.J. (2015). NOD-like receptors: Versatile cytosolic sentinels. Physiol. Rev..

[37] Fritz J.H., Ferrero R.L., Philpott D.J., Girardin S.E. (2006). Nod-like proteins in immunity, inflammation and disease. Nat. Immunol..

[38] Martinon F., Mayor A., Tschopp J. (2009). The Inflammasomes: Guardians of the Body. Annu. Rev. Immunol..

[39] Duéñez-Guzmán E.A., Haig D. (2014). The evolution of reproduction-related NLRP genes. J. Mol. Evol..

[40] McDaniel P., Wu X. (2009). Identification of oocyte-selective NLRP genes in rhesus macaque monkeys (Macaca mulatta). Mol. Reprod. Dev..

[41] Peng H., Chang B., Lu C., Su J., Wu Y., Lv P., Wang Y., Liu J., Zhang B., Quan F. (2012). Nlrp2, a maternal effect gene required for early embryonic development in the mouse. PLoS ONE.

[42] Wu X. (2009). Maternal depletion of NLRP5 blocks early embryogenesis in rhesus macaque monkeys (Macaca mulatta). Hum. Reprod..

[43] Zhang P., Dixon M., Zucchelli M., Hambiliki F., Levkov L., Hovatta O., Kere J. (2008). Expression analysis of the NLRP gene family suggests a role in human preimplantation development. PLoS ONE.

[44] Ogura Y., Bonen D.K., Inohara N., Nicolae D.L., Chen F.F., Ramos R., Britton H., Moran T., Karaliuskas R., Duerr R.H. (2001). A frameshift mutation in NOD2 associated with susceptibility to Crohn’s disease. Nature.

[45] Chang C.H., Guerder S., Hong S.C., Van Ewijk W., Flavell R.A. (1996). Mice lacking the MHC class II transactivator (CIITA) show tissue-specific impairment of MHC class II expression. Immunity.

[46] Staehli F., Ludigs K., Heinz L.X., Seguín-Estévez Q., Ferrero I., Braun M., Schroder K., Rebsamen M., Tardivel A., Mattmann C. (2012). NLRC5 Deficiency Selectively Impairs MHC Class I- Dependent Lymphocyte Killing by Cytotoxic T Cells. J. Immunol..

[47] Kofoed E.M., Vance R.E. (2011). Innate immune recognition of bacterial ligands by NAIPs determines inflammasome specificity. Nature.

[48] Lightfield K.L., Persson J., Brubaker S.W., Witte C.E., von Moltke J., Dunipace E.A., Henry T., Sun Y.H., Cado D., Dietrich W.F. (2008). Critical function for Naip5 in inflammasome activation by a conserved carboxy-terminal domain of flagellin. Nat. Immunol..

[49] Caruso R., Warner N., Inohara N., Núñez G. (2014). NOD1 and NOD2: Signaling, host defense, and inflammatory disease. Immunity.

[50] Kobayashi K., Inohara N., Hernandez L.D., Galán J.E., Núñez G., Janeway C.A., Medzhitov R., Flavell R.A. (2002). RICK/Rip2/CARDIAK mediates signalling for receptors of the innate and adaptive immune systems. Nature.

[51] Zambetti L.P., Laudisi F., Licandro G., Ricciardi-Castagnoli P., Mortellaro A. (2012). The rhapsody of NLRPs: Master players of inflammation… and a lot more. Immunol. Res..

[52] Martinon F., Pétrilli V., Mayor A., Tardivel A., Tschopp J. (2006). Gout-associated uric acid crystals activate the NALP3 inflammasome. Nature.

[53] Pazár B., Ea H.-K., Narayan S., Kolly L., Bagnoud N., Chobaz V., Roger T., Lioté F., So A., Busso N. (2011). Basic calcium phosphate crystals induce monocyte/macrophage IL-1β secretion through the NLRP3 inflammasome in vitro. J. Immunol..

[54] Salminen A., Ojala J., Suuronen T., Kaarniranta K., Kauppinen A. (2008). Amyloid-beta oligomers set fire to inflammasomes and induce Alzheimer’s pathology. J. Cell. Mol. Med..

[55] Wen H., Gris D., Lei Y., Jha S., Zhang L., Huang M.T.H., Brickey W.J., Ting J.P.Y. (2011). Fatty acid-induced NLRP3-ASC inflammasome activation interferes with insulin signaling. Nat. Immunol..

[56] Martinon F., Burns K., Tschopp J. (2002). The Inflammasome: A molecular platform triggering activation of inflammatory caspases and processing of proIL-β. Mol. Cell.

[57] Elinav E., Strowig T., Kau A.L., Henao-Mejia J., Thaiss C.A., Booth C.J., Peaper D.R., Bertin J., Eisenbarth S.C., Gordon J.I. (2011). NLRP6 inflammasome regulates colonic microbial ecology and risk for colitis. Cell.

[58] Khare S., Dorfleutner A., Bryan N.B., Yun C., Radian A.D., de Almeida L., Rojanasakul Y., Stehlik C. (2012). An NLRP7-containing inflammasome mediates recognition of microbial lipopeptides in human macrophages. Immunity.

[59] Vladimer G.I., Weng D., Paquette S.W.M., Vanaja S.K., Rathinam V.A.K., Aune M.H., Conlon J.E., Burbage J.J., Proulx M.K., Liu Q. (2012). The NLRP12 inflammasome recognizes Yersinia pestis. Immunity.

[60] Kufer T.A., Sansonetti P.J. (2011). NLR functions beyond pathogen recognition. Nat. Immunol..

[61] Duewell P., Kono H., Rayner K.J., Sirois C.M., Vladimer G., Bauernfeind F.G., Abela G.S., Franchi L., Nũez G., Schnurr M. (2010). NLRP3 inflammasomes are required for atherogenesis and activated by cholesterol crystals. Nature.

[62] Rajamäki K., Lappalainen J., Oörni K., Välimäki E., Matikainen S., Kovanen P.T., Eklund K.K. (2010). Cholesterol crystals activate the NLRP3 inflammasome in human macrophages: A novel link between cholesterol metabolism and inflammation. PLoS ONE.

[63] Hu Y., Lin H., Dib B., Atik A., Bouzika P., Lin C., Yan Y., Tang S., Miller J.W., Vavvas D.G. (2014). Cholesterol crystals induce inflammatory cytokines expression in a human retinal pigment epithelium cell line by activating the NF-κB pathway. Discov. Med..

[64] He Q., You H., Li X.M., Liu T.H., Wang P., Wang B.E. (2012). HMGB1 promotes the synthesis of pro-il-1β and pro-il-18 by activation of p38 MAPK and NF-kB through receptors for advanced glycation end-products in macrophages. Asian Pacific J. Cancer Prev..

[65] Weber M.D., Frank M.G., Tracey K.J., Watkins L.R., Maier S.F. (2015). Stress induces the danger-associated molecular pattern HMGB-1 in the hippocampus of male Sprague Dawley rats: A priming stimulus of microglia and the NLRP3 inflammasome. J. Neurosci..

[66] Chen X.-L., Zhang X.-D., Li Y.-Y., Chen X.-M., Tang D.-R., Ran R.-J. (2013). Involvement of HMGB1 mediated signalling pathway in diabetic retinopathy: Evidence from type 2 diabetic rats and ARPE-19 cells under diabetic condition. Br. J. Ophthalmol..

[67] Benko S., Tozser J., Miklossy G., Varga A., Kadas J., Csutak A., Berta A., Rajnavolgyi E. (2008). Constitutive and UV-B modulated transcription of NOD-like receptors and their functional partners in human corneal epithelial cells. Mol. Vis..

[68] Oh J.Y., Ko J.H., Ryu J.S., Lee H.J., Kim M.K., Wee W.R. (2017). Transcription Profiling of NOD-like receptors in the Human Cornea with Disease. Ocul. Immunol. Inflamm..

[69] Zhang Y., Wu J., Xin Z., Wu X. (2014). Aspergillus fumigatus triggers innate immune response via NOD1 signaling in human corneal epithelial cells. Exp. Eye Res..

[70] Wu J., Zhang Y., Xin Z., Wu X. (2015). The crosstalk between TLR2 and NOD2 in Aspergillus fumigatus keratitis. Mol. Immunol..

[71] Yang X., Zhao G., Yan J., Xu R., Che C., Zheng H., Zhu G., Zhang J. (2019). Pannexin 1 Channels Contribute to IL-1β Expression via NLRP3/Caspase-1 Inflammasome in Aspergillus Fumigatus Keratitis. Curr. Eye Res..

[72] McClellan S.A., Jerome A., Suvas S., Hazlett L.D. (2017). NLRC4 regulates caspase-1 and IL-1beta production in a CD11blowLy6Glow population of cells required for resistance to Pseudomonas aeruginosa keratitis. PLoS ONE.

[73] Karmakar M., Katsnelson M., Malak H.A., Greene N.G., Howell S.J., Hise A.G., Camilli A., Kadioglu A., Dubyak G.R., Pearlman E. (2015). Neutrophil IL-1β Processing Induced by Pneumolysin Is Mediated by the NLRP3/ASC Inflammasome and Caspase-1 Activation and Is Dependent on K + Efflux. J. Immunol..

[74] Shimizu H., Sakimoto T., Yamagami S. (2019). Pro-inflammatory role of NLRP3 inflammasome in experimental sterile corneal inflammation. Sci. Rep..

[75] Sun Y., Abbondante S., Karmakar M., de Jesus Carrion S., Che C., Hise A.G., Pearlman E. (2018). Neutrophil Caspase-11 Is Required for Cleavage of Caspase-1 and Secretion of IL-1β in Aspergillus fumigatus Infection. J. Immunol..

[76] Zheng Q., Ren Y., Reinach P.S., Xiao B., Lu H., Zhu Y., Qu J., Chen W. (2015). Reactive oxygen species activated NLRP3 inflammasomes initiate inflammation in hyperosmolarity stressed human corneal epithelial cells and environment-induced dry eye patients. Exp. Eye Res..

[77] Chi W., Hua X., Chen X., Bian F., Yuan X., Zhang L., Wang X., Chen D., Deng R., Li Z. (2017). Mitochondrial DNA oxidation induces imbalanced activity of NLRP3/NLRP6 inflammasomes by activation of caspase-8 and BRCC36 in dry eye. J. Autoimmun..

[78] Zheng Q., Ren Y., Reinach P.S., She Y., Xiao B., Hua S., Qu J., Chen W. (2014). Reactive oxygen species activated NLRP3 inflammasomes prime environment-induced murine dry eye. Exp. Eye Res..

[79] Bian F., Xiao Y., Zaheer M., Volpe E.A., Pflugfelder S.C., Li D.Q., De Paiva C.S. (2017). Inhibition of NLRP3 inflammasome pathway by butyrate improves corneal wound healing in corneal alkali burn. Int. J. Mol. Sci..

[80] Zheng Q., Tan Q., Ren Y., Reinach P.S., Li L., Ge C., Qu J., Chen W. (2018). Hyperosmotic stress–induced TRPM2 channel activation stimulates NLRP3 inflammasome activity in primary human corneal epithelial cells. Investig. Ophthalmol. Vis. Sci..

[81] Dai Y., Zhang J., Xiang J., Li Y., Wu D., Xu J. (2019). Calcitriol inhibits ROS-NLRP3-IL-1β signaling axis via activation of Nrf2-antioxidant signaling in hyperosmotic stress stimulated human corneal epithelial cells. Redox Biol..

[82] Rodríguez-Martínez S., Cancino-Díaz M.E., Jiménez-Zamudio L., García-Latorre E., Cancino-Díaz J.C. (2005). TLRs and NODs mRNA expression pattern in healthy mouse eye. Br. J. Ophthalmol..

[83] Del Mar Cendra M., Christodoulides M., Hossain P. (2017). Signaling mediated by Toll-Like receptor 5 sensing of Pseudomonas aeruginosa flagellin influences IL-1β and IL-18 production by primary fibroblasts derived from the human cornea. Front. Cell. Infect. Microbiol..

[84] Chaurasia S., Lim R., Lakshminarayanan R., Mohan R. (2015). Nanomedicine Approaches for Corneal Diseases. J. Funct. Biomater..

[85] Fukuda K., Ishida W., Fukushima A., Nishida T. (2017). Corneal fibroblasts as sentinel cells and local immune modulators in infectious keratitis. Int. J. Mol. Sci..

[86] Li Z., Wei C., Wang S., Liu T., Zhai H., Shi W. (2017). Upregulation of NLRP3 inflammasome components in Mooren’s ulcer. Graefe’s Arch. Clin. Exp. Ophthalmol..

[87] McGilligan V.E., Gregory-Ksander M.S., Li D., Moore J.E., Hodges R.R., Gilmore M.S., Moore T.C.B., Dartt D.A. (2013). Staphylococcus aureus Activates the NLRP3 Inflammasome in Human and Rat Conjunctival Goblet Cells. PLoS ONE.

[88] Sun N., Zhang H. (2018). Pyroptosis in pterygium pathogenesis. Biosci. Rep..

[89] Li D., Hodges R.R., Bispo P., Gilmore M.S., Gregory-Ksander M., Dartt D.A. (2017). Neither non-toxigenic Staphylococcus aureus nor commensal S. epidermidi activates NLRP3 inflammasomes in human conjunctival goblet cells. BMJ Open Ophthalmol..

[90] Scurrell E., Stanley R., Schöniger S. (2009). Immunohistochemical detection of NOD1 and NOD2 in the healthy murine and canine eye. Vet. Ophthalmol..

[91] Chi W., Li F., Chen H., Wang Y., Zhu Y., Yang X., Zhu J., Wu F., Ouyang H., Ge J. (2014). Caspase-8 promotes NLRP1/NLRP3 inflammasome activation and IL-1β production in acute glaucoma. Proc. Natl. Acad. Sci. USA.

[92] Zhu J., Chen L., Qi Y., Feng J., Zhu L., Bai Y., Wu H. (2018). Protective effects of Erigeron breviscapus Hand.-Mazz. (EBHM) extract in retinal neurodegeneration models. Mol. Vis..

[93] Albalawi F., Lu W., Beckel J.M., Lim J.C., McCaughey S.A., Mitchell C.H. (2017). The P2 × 7 receptor primes IL-1β and the NLRP3 inflammasome in astrocytes exposed to mechanical strain. Front. Cell. Neurosci..

[94] Pronin A., Pham D., An W., Dvoriantchikova G., Reshetnikova G., Qiao J., Kozhekbaeva Z., Reiser A.E., Slepak V.Z., Shestopalov V.I. (2019). Inflammasome activation induces pyroptosis in the retina exposed to ocular hypertension injury. Front. Mol. Neurosci..

[95] Qi Y., Zhao M., Bai Y., Huang L., Yu W., Bian Z., Zhao M., Li X. (2014). Retinal ischemia/reperfusion injury is mediated by tolllike receptor 4 activation of NLRP3 inflammasomes. Investig. Ophthalmol. Vis. Sci..

[96] Qijun Z., Huan Z., Ling G., Kaijian C., Wei L., Shuxing J., Xiang C., Rongdi Y., Jian Y. (2019). The levels and significance of inflammasomes in the mouse retina following optic nerve crush. Int. Immunopharmacol..

[97] Puyang Z., Feng L., Chen H., Liang P., Troy J.B., Liu X. (2016). Retinal ganglion cell loss is delayed following optic nerve crush in nlrp3 knockout mice. Sci. Rep..

[98] Harun-Or-Rashid M., Inman D.M. (2018). Reduced AMPK activation and increased HCAR activation drive anti-inflammatory response and neuroprotection in glaucoma. J. Neuroinflammation.

[99] Xu Z., Fouda A.Y., Lemtalsi T., Shosha E., Rojas M., Liu F., Patel C., Caldwell R.W., Narayanan S.P., Caldwell R.B. (2018). Retinal Neuroprotection from Optic Nerve Trauma by Deletion of Arginase 2. Front. Neurosci..

[100] Chi W., Chen H., Li F., Zhu Y., Yin W., Zhuo Y. (2015). HMGB1 promotes the activation of NLRP3 and caspase-8 inflammasomes via NF-κB pathway in acute glaucoma. J. Neuroinflammation.

[101] Lin C., Wu F., Zheng T., Wang X., Chen Y., Wu X. (2019). Kaempferol attenuates retinal ganglion cell death by suppressing NLRP1/NLRP3 inflammasomes and caspase-8 via JNK and NF-κB pathways in acute glaucoma. Eye.

[102] Lei X., Zhao Y. (2019). Neovascular glaucoma regulation by arylsulfonyl indoline-benzamide (ASIB) through targeting NF-kB signalling pathway. 3 Biotech..

[103] Hu Z., Zhang Y., Wang J., Mao P., Lv X., Yuan S., Huang Z., Ding Y., Xie P., Liu Q. (2016). Knockout of Ccr2 alleviates photoreceptor cell death in rodent retina exposed to chronic blue light. Cell Death Dis..

[104] Jiang D., Ryals R.C., Huang S.J., Weller K.K., Titus H.E., Robb B.M., Saad F.W., Salam R.A., Hammad H., Yang P. (2019). Monomethyl fumarate protects the retina from light- induced retinopathy. Investig. Ophthalmol. Vis. Sci..

[105] Tsoka P., Barbisan P.R., Kataoka K., Chen X.N., Tian B., Bouzika P., Miller J.W., Paschalis E.I., Vavvas D.G. (2019). NLRP3 inflammasome in NMDA-induced retinal excitotoxicity. Exp. Eye Res..

[106] El-Azab M.F., Baldowski B.R.B., Mysona B.A., Shanab A.Y., Mohamed I.N., Abdelsaid M.A., Matragoon S., Bollinger K.E., Saul A., El-Remessy A.B. (2014). Deletion of thioredoxin-interacting protein preserves retinal neuronal function by preventing inflammation and vascular injury. Br. J. Pharmacol..

[107] Viringipurampeer I.A., Metcalfe A.L., Bashar A.E., Sivak O., Yanai A., Mohammadi Z., Moritz O.L., Gregory-Evans C.Y., Gregory-Evans K. (2016). NLRP3 inflammasome activation drives bystander cone photoreceptor cell death in a P23H rhodopsin model of retinal degeneration. Hum. Mol. Genet..

[108] Appelbaum T., Santana E., Aguirre G.D. (2017). Strong upregulation of inflammatory genes accompanies photoreceptor demise in canine models of retinal degeneration. PLoS ONE.

[109] Li Y., Liu C., Wan X.S., Li S.W. (2018). NLRP1 deficiency attenuates diabetic retinopathy (DR) in mice through suppressing inflammation response. Biochem. Biophys. Res. Commun..

[110] Wang S., Ji L.Y., Li L., Li J.M. (2019). Oxidative stress, autophagy and pyroptosis in the neovascularization of oxygen-induced retinopathy in mice. Mol. Med. Rep..

[111] Rivera J.C., Sitaras N., Noueihed B., Hamel D., Madaan A., Zhou T., Honoré J.-C., Quiniou C., Joyal J.-S., Hardy P. (2013). Microglia and interleukin-1β in ischemic retinopathy elicit microvascular degeneration through neuronal semaphorin-3A. Arterioscler. Thromb. Vasc. Biol..

[112] Davey M.P., Martin T.M., Planck S.R., Lee J., Zamora D., Rosenbaum J.T. (2006). Human endothelial cells express NOD2/CARD15 and increase IL-6 secretion in response to muramyl dipeptide. Microvasc. Res..

[113] Mohamed I.N., Hafez S.S., Fairaq A., Ergul A., Imig J.D., El-Remessy A.B. (2014). Thioredoxin-interacting protein is required for endothelial NLRP3 inflammasome activation and cell death in a rat model of high-fat diet. Diabetologia.

[114] Chaurasia S.S., Lim R.R., Parikh B.H., Yeo S.W., Tun B.B., Wong T.Y., Luu C.D., Agrawal R., Ghosh A., Mortellaro A. (2018). The NLRP3 Inflammasome May Contribute to Pathologic Neovascularization in the Advanced Stages of Diabetic Retinopathy. Sci. Rep..

[115] Liu R.T., Gao J., Cao S., Sandhu N., Cui J.Z., Chou C.L., Fang E., Matsubara J.A. (2013). Inflammatory mediators induced by amyloid-beta in the retina and RPE in vivo: Implications for inflammasome activation in age-related macular degeneration. Invest. Ophthalmol. Vis. Sci..

[116] Lei C., Lin R., Wang J., Tao L., Fu X., Qiu Y., Lei B. (2017). Amelioration of amyloid β-induced retinal inflammatory responses by a LXR agonist TO901317 is associated with inhibition of the NF-κB signaling and NLRP3 inflammasome. Neuroscience.

[117] Wang Y., Hanus J.W., Abu-Asab M.S., Shen D., Ogilvy A., Ou J., Chu X.K., Shi G., Li W., Wang S. (2016). NLRP3 upregulation in retinal pigment epithelium in age-related macular degeneration. Int. J. Mol. Sci..

[118] Aredo B., Li T., Chen X., Zhang K., Wang C.X.Z., Gou D., Zhao B., He Y., Ufret-Vincenty R.L. (2015). A chimeric Cfh transgene leads to increased retinal oxidative stress, inflammation, and accumulation of activated subretinal microglia in mice. Investig. Ophthalmol. Vis. Sci..

[119] Basu S., Fowler B.J., Kerur N., Arnvig K.B., Rao N.A. (2018). NLRP3 inflammasome activation by mycobacterial ESAT-6 and dsRNA in intraocular tuberculosis. Microb. Pathog..

[120] Zhang Y., Lv X., Hu Z., Ye X., Zheng X., Ding Y., Xie P., Liu Q. (2017). Protection of Mcc950 against high-glucose-induced human retinal endothelial cell dysfunction. Cell Death Dis..

[121] Li S., Yang H., Chen X. (2019). Protective effects of sulforaphane on diabetic retinopathy: Activation of the Nrf2 pathway and inhibition of NLRP3 inflammasome formation. Exp. Anim..

[122] Hao J., Zhang H., Yu J., Chen X., Yang L. (2018). Methylene Blue Attenuates Diabetic Retinopathy by Inhibiting NLRP3 Inflammasome Activation in STZ-induced Diabetic Rats. Ocul. Immunol. Inflamm..

[123] Liu Q., Zhang F., Zhang X., Cheng R., Ma J.X., Yi J., Li J. (2018). Fenofibrate ameliorates diabetic retinopathy by modulating Nrf2 signaling and NLRP3 inflammasome activation. Mol. Cell. Biochem..

[124] Indaram M., Ma W., Zhao L., Fariss R.N., Rodriguez I.R., Wong W.T. (2015). 7-Ketocholesterol Increases Retinal Microglial Migration, Activation, and Angiogenicity: A Potential Pathogenic Mechanism Underlying Age-related Macular Degeneration. Sci. Rep..

[125] Devi T.S., Lee I., Hüttemann M., Kumar A., Nantwi K.D., Singh L.P. (2012). TXNIP links innate host defense mechanisms to oxidative stress and inflammation in retinal muller glia under chronic hyperglycemia: Implications for diabetic retinopathy. Exp. Diabetes Res..

[126] Coucha M., Mohamed I.N., Elshaer S.L., Mbata O., Bartasis M.L., El-Remessy A.B. (2017). High fat diet dysregulates microRNA-17-5p and triggers retinal inflammation: Role of endoplasmic-reticulum-stress. World J. Diabetes.

[127] Hu L., Yang H., Ai M., Jiang S. (2017). Inhibition of TLR4 alleviates the inflammation and apoptosis of retinal ganglion cells in high glucose. Graefe’s Arch. Clin. Exp. Ophthalmol..

[128] Jiang Y., Liu L., Curtiss E., Steinle J.J. (2017). Epac1 Blocks NLRP3 Inflammasome to Reduce IL-1 β in Retinal Endothelial Cells and Mouse Retinal Vasculature. Mediators Inflamm..

[129] Tseng W.A., Thein T., Kinnunen K., Lashkari K., Gregory M.S., D’Amore P.A., Ksander B.R. (2013). NLRP3 inflammasome activation in retinal pigment epithelial cells by lysosomal destabilization: Implications for age-related macular degeneration. Invest. Ophthalmol. Vis. Sci..

[130] Tarallo V., Hirano Y., Gelfand B.D., Dridi S., Kerur N., Kim Y., Cho W.G., Kaneko H., Fowler B.J., Bogdanovich S. (2012). DICER1 loss and Alu RNA induce age-related macular degeneration via the NLRP3 inflammasome and MyD88. Cell.

[131] Liu R.T., Wang A., To E., Gao J., Cao S., Cui J.Z., Matsubara J.A. (2014). Vinpocetine inhibits amyloid-beta induced activation of NF-κB, NLRP3 inflammasome and cytokine production in retinal pigment epithelial cells. Exp. Eye Res..

[132] Gelfand B.D., Wright C.B., Kim Y., Yasuma T., Yasuma R., Li S., Fowler B.J., Bastos-Carvalho A., Kerur N., Uittenbogaard A. (2015). Iron toxicity in the retina requires Alu RNA and the NLRP3 inflammasome. Cell Rep..

[133] Marneros A. (2013). NLRP3 inflammasome blockade inhibits VEGF-A-induced age-related macular degeneration. Cell Rep..

[134] Prager P., Hollborn M., Steffen A., Wiedemann P., Kohen L., Bringmann A. (2016). P2Y1 receptor signaling contributes to high salt-induced priming of the NLRP3 inflammasome in retinal pigment epithelial cells. PLoS ONE.

[135] Hollborn M., Ackmann C., Kuhrt H., Doktor F., Kohen L., Wiedemann P., Bringmann A. (2018). Osmotic and hypoxic induction of the complement factor C9 in cultured human retinal pigment epithelial cells: Regulation of VEGF and NLRP3 expression. Mol. Vis..

[136] Zhang S., Yu N., Zhang R., Zhang S., Wu J. (2016). Interleukin-17A induces IL-1β secretion from RPE cells via the NLRP3 inflammasome. Investig. Ophthalmol. Vis. Sci..

[137] Kerur N., Hirano Y., Tarallo V., Fowler B.J., Bastos-Carvalho A., Yasuma T., Yasuma R., Kim Y., Hinton D.R., Kirschning C.J. (2013). TLR-independent and P2 × 7-dependent signaling mediate Alu RNA-induced NLRP3 inflammasome activation in geographic atrophy. Investig. Ophthalmol. Vis. Sci..

[138] Gnanaguru G., Choi A.R., Amarnani D., D’Amore P.A. (2016). Oxidized lipoprotein uptake through the CD36 receptor activates the NLRP3 inflammasome in human retinal pigment epithelial cells. Investig. Ophthalmol. Vis. Sci..

[139] Jin X., Wang C., Wu W., Liu T., Ji B., Zhou F. (2018). Cyanidin-3-glucoside alleviates 4-Hydroxyhexenal-induced NLRP3 inflammasome activation via JNK-c-Jun/AP-1 pathway in human retinal pigment epithelial cells. J. Immunol. Res..

[140] Cao S., Wang J.C.C., Gao J., Wong M., To E., White V.A., Cui J.Z., Matsubara J.A. (2016). CFH Y402H polymorphism and the complement activation product C5a: Effects on NF-κB activation and inflammasome gene regulation. Br. J. Ophthalmol..

[141] Piippo N., Korkmaz A., Hytti M., Kinnunen K., Salminen A., Atalay M., Kaarniranta K., Kauppinen A. (2014). Decline in cellular clearance systems induces inflammasome signaling in human ARPE-19 cells. Biochim. Biophys. Acta Mol. Cell Res..

[142] Mao X., Pan T., Shen H., Xi H., Yuan S., Liu Q. (2018). The rescue effect of mesenchymal stem cell on sodium iodate-induced retinal pigment epithelial cell death through deactivation of NF-κB-mediated NLRP3 inflammasome. Biomed. Pharmacother..

[143] Mugisho O.O., Green C.R., Kho D.T., Zhang J., Graham E.S., Acosta M.L., Rupenthal I.D. (2018). The inflammasome pathway is amplified and perpetuated in an autocrine manner through connexin43 hemichannel mediated ATP release. Biochim. Biophys. Acta Gen. Subj..

[144] Wang K., Zhu X., Zhang K., Yao Y., Zhuang M., Tan C., Zhou F., Zhu L. (2017). Puerarin inhibits amyloid β-induced NLRP3 inflammasome activation in retinal pigment epithelial cells via suppressing ROS-dependent oxidative and endoplasmic reticulum stresses. Exp. Cell Res..

[145] Mao K. (2019). Salvianolic Acid A Protects Retinal Pigment Epithelium from O… Salvianolic Acid A Protects Retinal Pigment Epithelium from O. Discov. Med..

[146] Wang K., Yao Y., Zhu X., Zhang K., Zhou F., Zhu L. (2017). Amyloid β induces NLRP3 inflammasome activation in retinal pigment epithelial cells via NADPH oxidase- and mitochondria-dependent ROS production. J. Biochem. Mol. Toxicol..

[147] Shi H., Zhang Z., Wang X., Li R., Hou W., Bi W., Zhang X. (2015). Inhibition of autophagy induces IL-1β release from ARPE-19 cells via ROS mediated NLRP3 inflammasome activation under high glucose stress. Biochem. Biophys. Res. Commun..

[148] Zhang W., Ma Y., Zhang Y., Yang J., He G., Chen S. (2019). Photo-Oxidative Blue-Light Stimulation in Retinal Pigment Epithelium Cells Promotes Exosome Secretion and Increases the Activity of the NLRP3 Inflammasome. Curr. Eye Res..

[149] Piippo N., Korhonen E., Hytti M., Skottman H., Kinnunen K., Josifovska N., Petrovski G., Kaarniranta K., Kauppinen A. (2018). Hsp90 inhibition as a means to inhibit activation of the NLRP3 inflammasome. Sci. Rep..

[150] Gao J., Liu R.T., Cao S., Cui J.Z., Wang A., To E., Matsubara J.A. (2015). NLRP3 Inflammasome: Activation and Regulation in Age-Related Macular Degeneration. Mediators Inflamm..

[151] Doyle S.L., Campbell M., Ozaki E., Salomon R.G., Mori A., Kenna P.F., Farrar G.J., Kiang A.S., Humphries M.M., Lavelle E.C. (2012). NLRP3 has a protective role in age-related macular degeneration through the induction of IL-18 by drusen components. Nat. Med..

[152] Anderson O.A., Finkelstein A., Shima D.T. (2013). A2E Induces IL-1ß Production in Retinal Pigment Epithelial Cells via the NLRP3 Inflammasome. PLoS ONE.

[153] Kowluru R.A., Kowluru A., Mishra M., Kumar B. (2015). Oxidative stress and epigenetic modifications in the pathogenesis of diabetic retinopathy. Prog. Retin. Eye Res..

[154] Kanwar M., Chan P.S., Kern T.S., Kowluru R.A. (2007). Oxidative damage in the retinal mitochondria of diabetic mice: Possible protection by superoxide dismutase. Investig. Ophthalmol. Vis. Sci..

[155] Goto H., Nishikawa T., Sonoda K., Kondo T., Kukidome D., Fujisawa K., Yamashiro T., Motoshima H., Matsumura T., Tsuruzoe K. (2008). Endothelial MnSOD overexpression prevents retinal VEGF expression in diabetic mice. Biochem. Biophys. Res. Commun..

[156] Kowluru R.A., Atasi L., Ho Y.S. (2006). Role of mitochondrial superoxide dismutase in the development of diabetic retinopathy. Investig. Ophthalmol. Vis. Sci..

[157] Lorenzi M. (2007). The polyol pathway as a mechanism for diabetic retinopathy: Attractive, elusive, and resilient. Exp. Diabetes Res..

[158] Kowluru R.A., Odenbach S. (2004). Role of interleukin-1β in the development of retinopathy in rats: Effect of antioxidants. Investig. Ophthalmol. Vis. Sci..

[159] Kirk S.L., Karlik S.J. (2003). VEGF and vascular changes in chronic neuroinflammation. J. Autoimmun..

[160] Rangasamy S., McGuire P.G., Franco Nitta C., Monickaraj F., Oruganti S.R., Das A. (2014). Chemokine mediated monocyte trafficking into the retina: Role of inflammation in alteration of the blood-retinal barrier in diabetic retinopathy. PLoS ONE.

[161] Joussen A.M., Poulaki V., Le M.L., Koizumi K., Esser C., Janicki H., Schraermeyer U., Kociok N., Fauser S., Kirchhof B. (2004). A central role for inflammation in the pathogenesis of diabetic retinopathy. FASEB J..

[162] Vincent J.A., Mohr S. (2007). Inhibition of caspase-1/interleukin-1beta signaling prevents degeneration of retinal capillaries in diabetes and galactosemia. Diabetes.

[163] Xie T.X., Xia Z., Zhang N., Gong W., Huang S. (2010). Constitutive NF-κB activity regulates the expression of VEGF and IL-8 and tumor angiogenesis of human glioblastoma. Oncol. Rep..

[164] Zheng L., Szabó C., Kern T.S. (2004). Poly(ADP-ribose) polymerase is involved in the development of diabetic retinopathy via regulation of nuclear factor-κB. Diabetes.

[165] Drel V.R., Xu W., Zhang J., Kador P.F., Ali T.K., Shin J., Julius U., Slusher B., El-Remessy A.B., Obrosova I.G. (2009). Poly(ADP-ribose)polymerase inhibition counteracts cataract formation and early retinal changes in streptozotocin-diabetic rats. Investig. Ophthalmol. Vis. Sci..

[166] Bauernfeind F.G., Horvath G., Stutz A., Alnemri E.S., Macdonald K., Speert D., Fernandes-Alnemri T., Monks B.G., Wu J., Fitzgerald K.A. (2009). Cutting Edge: NF-κ B Activating Pattern Recognition and Cytokine Receptors License NLRP3 Inflammasome Activation by Regulating NLRP3 Expression. J. Immunol..

[167] Qiao Y., Wang P., Qi J., Zhang L., Gao C. (2012). TLR-induced NF-κB activation regulates NLRP3 expression in murine macrophages. FEBS Lett..

[168] Tukhvatulin A.I., Logunov D.Y., Gitlin I.I., Shmarov M.M., Kudan P.V., Adzhieva C.A.C.A., Moroz A.F., Kostyukova N.N., Burdelya L.G., Naroditsky B.S. (2011). A In Vitro and In Vivo Study of the Ability of NOD1 Ligands to Activate the Transcriptional Factor NF-kB. Acta Naturae.

[169] Ting J.P.Y., Duncan J.A., Lei Y. (2010). How the noninflammasome NLRs function in the innate immune system. Science.

[170] Fontalba A., Gutierrez O., Fernandez-Luna J.L. (2007). NLRP2, an inhibitor of the NF-kappaB pathway, is transcriptionally activated by NF-kappaB and exhibits a nonfunctional allelic variant. J. Immunol..

[171] Anand P.K., Malireddi R.K.S., Lukens J.R., Vogel P., Bertin J., Lamkanfi M., Kanneganti T.-D. (2012). NLRP6 negatively regulates innate immunity and host defence against bacterial pathogens. Nature.

[172] Chen G.Y., Liu M., Wang F., Bertin J., Núñez G. (2011). A functional role for Nlrp6 in intestinal inflammation and tumorigenesis. J. Immunol..

[173] Schneider M., Zimmermann A.G., Roberts R.A., Zhang L., Swanson K.V., Wen H., Davis B.K., Allen I.C., Holl E.K., Ye Z. (2012). The innate immune sensor NLRC3 attenuates Toll-like receptor signaling via modification of the signaling adaptor TRAF6 and transcription factor NF-κB. Nat. Immunol..

[174] Allen I.C., Wilson J.E., Schneider M., Lich J.D., Roberts R.A., Arthur J.C., Woodford R.M.T., Davis B.K., Uronis J.M., Herfarth H.H. (2012). NLRP12 Suppresses Colon Inflammation and Tumorigenesis through the Negative Regulation of Noncanonical NF-κB Signaling. Immunity.

[175] Moore C.B., Bergstralh D.T., Duncan J.A., Lei Y., Morrison T.E., Zimmermann A.G., Accavitti-Loper M.A., Madden V.J., Sun L., Ye Z. (2008). NLRX1 is a regulator of mitochondrial antiviral immunity. Nature.

[176] Lupfer C., Kanneganti T.-D. (2013). Unsolved Mysteries in NLR Biology. Front. Immunol..

[177] Kawai M., Yoshikawa T., Nishikomori R., Heike T., Takahashi K. (2013). Obvious optic disc swelling in a patient with cryopyrin-associated periodic syndrome. Clin. Ophthalmol..

[178] Alejandre N., Ruiz-Palacios A., García-Aparicio A.M., Blanco-Kelly F., Bermúdez S., Fernández-Sanz G., Romero F.I., Aróstegui J.I., Ayuso C., Jiménez-Alfaro I. (2014). Description of a new family with cryopyrin-associated periodic syndrome: Risk of visual loss in patients bearing the R260W mutation. Rheumatol. (United Kingdom).

[179] Oberg T.J., Vitale A.T., Hoffman R.O., Bohnsack J.F., Warner J.E. (2013). Cryopyrin-associated periodic syndromes and the eye. Ocul. Immunol. Inflamm..

[180] Mohr S., Xi A., Tang J., Kern T.S. (2002). Caspase activation in retinas of diabetic and galactosemic mice and diabetic patients. Diabetes.

[181] El Asrar A.M.A., Maimone D., Morse P.H., Gregory S., Reder A.T. (1992). Cytokines in the vitreous of patients with proliferative diabetic retinopathy. Am. J. Ophthalmol..

[182] Feenstra D.J., Yego E.C., Mohr S. (2013). Modes of Retinal Cell Death in Diabetic Retinopathy. J. Clin. Exp. Ophthalmol..

[183] Kowluru R.A. (2019). Mitochondrial stability in diabetic retinopathy: Lessons learned from epigenetics. Diabetes.

[184] Shimada K., Crother T.R., Karlin J., Dagvadorj J., Chiba N., Chen S., Ramanujan V.K., Wolf A.J., Vergnes L., Ojcius D.M. (2012). Oxidized Mitochondrial DNA Activates the NLRP3 Inflammasome during Apoptosis. Immunity.

[185] Liu Q., Zhang D., Hu D., Zhou X., Zhou Y. (2018). The role of mitochondria in NLRP3 inflammasome activation. Mol. Immunol..

[186] Singh L.P., Yumnamcha T., Swornalata Devi T. (2018). Mitophagic Flux Deregulation, Lysosomal Destabilization and NLRP3 Inflammasome Activation in Diabetic Retinopathy: Potentials of Gene Therapy Targeting TXNIP and The Redox System. Ophthalmol. Res. reports.

[187] McGuire K.A., Barlan A.U., Griffin T.M., Wiethoff C.M. (2011). Adenovirus Type 5 Rupture of Lysosomes Leads to Cathepsin B-Dependent Mitochondrial Stress and Production of Reactive Oxygen Species. J. Virol..

[188] Klettner A., Kauppinen A., Blasiak J., Roider J., Salminen A., Kaarniranta K. (2013). Cellular and molecular mechanisms of age-related macular degeneration: From impaired autophagy to neovascularization. Int. J. Biochem. Cell Biol..

[189] Nakahira K., Haspel J.A., Rathinam V.A.K., Lee S.J., Dolinay T., Lam H.C., Englert J.A., Rabinovitch M., Cernadas M., Kim H.P. (2011). Autophagy proteins regulate innate immune responses by inhibiting the release of mitochondrial DNA mediated by the NALP3 inflammasome. Nat. Immunol..

[190] Perrone L., Devi T.S., Hosoya K., Terasaki T., Singh L.P. (2009). Thioredoxin interacting protein (TXNIP) induces inflammation through chromatin modification in retinal capillary endothelial cells under diabetic conditions. J. Cell. Physiol..

[191] Devi T.S., Hosoya K.-I., Terasaki T., Singh L.P. (2013). Critical role of TXNIP in oxidative stress, DNA damage and retinal pericyte apoptosis under high glucose: Implications for diabetic retinopathy. Exp. Cell Res..

[192] Parikh H., Carlsson E., Chutkow W.A., Johansson L.E., Storgaard H., Poulsen P., Saxena R., Ladd C., Schulze P.C., Mazzini M.J. (2007). TXNIP regulates peripheral glucose metabolism in humans. PLoS Med..

[193] Zhou R., Tardivel A., Thorens B., Choi I., Tschopp J. (2010). Thioredoxin-interacting protein links oxidative stress to inflammasome activation. Nat. Immunol..

[194] Trueblood K.E., Mohr S., Dubyak G.R. (2011). Purinergic regulation of high-glucose-induced caspase-1 activation in the rat retinal Müller cell line rMC-1. Am. J. Physiol. Cell Physiol..

[195] Devi T.S., Somayajulu M., Kowluru R.A., Singh L.P. (2017). TXNIP regulates mitophagy in retinal Müller cells under high-glucose conditions: Implications for diabetic retinopathy. Cell Death Dis..

[196] Schulze P.C., Yoshioka J., Takahashi T., He Z., King G.L., Lee R.T. (2004). Hyperglycemia promotes oxidative stress through inhibition of thioredoxin function by thioredoxin-interacting protein. J. Biol. Chem..

[197] Perrone L., Devi T.S., Hosoya K.-I., Terasaki T., Singh L.P. (2010). Inhibition of TXNIP expression in vivo blocks early pathologies of diabetic retinopathy. Cell Death Dis..

[198] Rashid K., Akhtar-Schaefer I., Langmann T. (2019). Microglia in Retinal Degeneration. Front. Immunol..

[199] Zeng H.Y., Green W.R., Tso M.O.M. (2008). Microglial activation in human diabetic retinopathy. Arch. Ophthalmol..

[200] Vujosevic S., Bini S., Midena G., Berton M., Pilotto E., Midena E. (2013). Hyperreflective intraretinal spots in diabetics without and with nonproliferative diabetic retinopathy: An in vivo study using spectral domain OCT. J. Diabetes Res..

[201] Zeng X.X., Ng Y.K., Ling E.A. (2000). Neuronal and microglial response in the retina of streptozotocin-induced diabetic rats. Vis. Neurosci..

[202] Lim R.R., Hainsworth D.P., Mohan R.R., Chaurasia S.S. (2019). Characterization of a functionally active primary microglial cell culture from the pig retina. Exp. Eye Res..

[203] Krady J.K., Basu A., Allen C.M., Xu Y., LaNoue K.F., Gardner T.W., Levison S.W. (2005). Minocycline reduces proinflammatory cytokine expression, microglial activation, and caspase-3 activation in a rodent model of diabetic retinopathy. Diabetes.

[204] Mangan M.S.J., Olhava E.J., Roush W.R., Seidel H.M., Glick G.D., Latz E. (2018). Targeting the NLRP3 inflammasome in inflammatory diseases. Nat. Rev. Drug Discov..

[205] Liao Y., Zhang H., He D., Wang Y., Cai B., Chen J., Ma J., Liu Z., Wu Y. (2019). Retinal pigment epithelium cell death is associated with NLRP3 inflammasome activation by all-trans retinal. Investig. Ophthalmol. Vis. Sci..

[206] Yumnamcha T., Devi T.S., Singh L.P. (2019). Auranofin Mediates Mitochondrial Dysregulation and Inflammatory Cell Death in Human Retinal Pigment Epithelial Cells: Implications of Retinal Neurodegenerative Diseases. Front. Neurosci..

[207] Lim R.R., Grant D.G., Olver T.D., Padilla J., Czajkowski A.M., Schnurbusch T.R., Mohan R.R., Hainsworth D.P., Walters E.M., Chaurasia S.S. (2018). Young Ossabaw Pigs Fed a Western Diet Exhibit Early Signs of Diabetic Retinopathy. Investig. Ophthalmol. Vis. Sci..

[208] Masland R.H. (2011). Cell populations of the retina: The Proctor lecture. Invest. Ophthalmol. Vis. Sci..

[209] Masland R.H. (2001). The fundamental plan of the retina. Nat. Neurosci..

